# Managing nitrogen in maize production for societal gain

**DOI:** 10.1093/pnasnexus/pgad319

**Published:** 2023-10-24

**Authors:** Andrew L Goodkind, Sumil K Thakrar, Stephen Polasky, Jason D Hill, David Tilman

**Affiliations:** Department of Economics, University of New Mexico, Albuquerque, NM 87131, USA; Department of Bioproducts and Biosystems Engineering, University of Minnesota, St. Paul, MN 55108, USA; Department of Applied Economics, University of Minnesota, St. Paul, MN 55108, USA; Department of Applied Economics, University of Minnesota, St. Paul, MN 55108, USA; Department of Bioproducts and Biosystems Engineering, University of Minnesota, St. Paul, MN 55108, USA; Department of Ecology, Evolution, and Behavior, University of Minnesota, St.Paul, MN 55108, USA

**Keywords:** environmental economics, air pollution, environmental science, agricultural economics, pollution costs

## Abstract

Highly productive agriculture is essential to feed humanity, but agricultural practices often harm human health and the environment. Using a nitrogen (N) mass-balance model to account for N inputs and losses to the environment, along with empirical based models of yield response, we estimate the potential gains to society from improvements in nitrogen management that could reduce health and environmental costs from maize grown in the US Midwest. We find that the monetized health and environmental costs to society of current maize nitrogen management practices are six times larger than the profits earned by farmers. Air emissions of ammonia from application of synthetic fertilizer and manure are the largest source of pollution costs. We show that it is possible to reduce these costs by 85% ($21.6 billion per year, 2020$) while simultaneously increasing farmer profits. These gains come from (i) managing fertilizer ammonia emissions by changing the mix of fertilizer and manure applied, (ii) improving production efficiency by reducing fertilization rates, and (iii) halting maize production on land where health and environmental costs exceed farmer profits, namely on low-productivity land and locations in which emissions are especially harmful. Reducing ammonia emissions from changing fertilizer types—in (i)—reduces health and environmental costs by 46% ($11.7 billion). Reducing fertilization rates—in (ii)—limits nitrous oxide emissions, further reducing health and environmental costs by $9.5 billion, and halting production on 16% of maize-growing land in the Midwest—in (iii)—reduces costs by an additional $0.4 billion.

**Significance Statement**

Agricultural practices produce food, animal feed, and biofuels, but also harm human health and the environment. In the US Midwest, current maize production results in nitrogen-related health and environmental costs that are six times larger than the profits earned by farmers. Changes in farming practices can substantially reduce these impacts while maintaining or increasing profits for farmers. These changes involve changing the form or method of application of nitrogen fertilizer, reducing fertilizer rates, and shifting production away from areas of low productivity or high environmental and health costs. These changes in farming practices, when spread over millions of hectares, can yield large societal gains. Our modeling approach could be applied to other regions and crops.

## Introduction

Accounting for health and environmental costs in agricultural production can lead to better decisions about what crops to produce and how and where to produce them. Here, we illustrate this potential by analyzing health and environmental costs of nitrogen (N) management in maize production in the United States (US) Midwest.

Maize production in the US Midwest is a globally significant agricultural sector, accounting for ∼20% of global maize output and ∼60% of US maize output. Current farming practices involve applying quantities of synthetic N fertilizers and manure that lead to substantial N losses to the environment. While N amendments are vital for agricultural production, N uptake for N-intensive crops is often <50% of the N applied (1, 2). When the amount of N applied exceeds N uptake, some fraction of the remaining N generates nitrous oxide (N_2_O) that contributes to climate change and ozone depletion (3, 4), ammonia (NH_3_) and nitrogen oxide (NO) that contribute to the formation of fine particulate matter (PM_2.5_), which is injurious to human health (5–7), nitrate (${\mathrm{NO}}_{3}^{-}$) that affects surface and ground water quality and human health (8), and non-harmful nitrogen gas (N_2_). Pollution costs from N fertilizer application depend on local soil and climatic conditions (9, 10), the volatilization rate of ammonia and other gases, and the magnitude of the human and ecosystem exposure to the N pollution along its biogeochemical pathway (11).

Several previous studies have examined the costs and benefits related to N management (2, 12–17). Our study expands on these studies by taking account of a wider range of pollution costs from various types of N fertilizer, including air pollution costs that make up a large portion of the estimated damages, and have previously been regarded as less consequential than the water quality impacts of N losses. We also account for spatial variability of air pollution costs, using an air-quality model that isolates the impact of emissions from specific source locations, which has only been considered to a limited extent in prior work (12, 16, 17). Prior work has not included a comprehensive estimate including the benefits of crop production from farmer profits and consumer surplus along with the health and environmental costs to show the relative magnitudes and tradeoffs involved. By incorporating location-specific air pollution costs into the decision-making process for N management, we find heterogeneous outcomes with larger reductions in N use and maize production in areas nearby and upwind of larger population centers.

Here, we quantify farmer profits and nitrogen-related health and environmental costs of maize production at the county level for the years 2013–22 in the US Midwest using a N mass-balance model accounting for N inputs and losses to the environment. We then evaluate the costs and benefits to farmers, consumers, and society of three changes from current practices as follows: (i) limiting ammonia loss to the atmosphere from N fertilizer by changing fertilization practices; (ii) lowering N fertilizer input in addition to limiting ammonia losses; and (iii) retiring land from maize production in the counties where health and environmental costs exceed farmer profits in addition to (i) and (ii). These changes are based on the current paradigm of known and adopted farming practices, and do not preclude the possibility of more impactful, transformative changes. However, the implementation of the changes at the scale we propose would require overcoming difficult obstacles including political barriers, concerns over price increases for consumers, consequences for rural farming communities that may retire farmland, and buy-in from key stakeholders.

## Results

### Pollution costs and farmer profits from current maize production

**Fig. 1.**
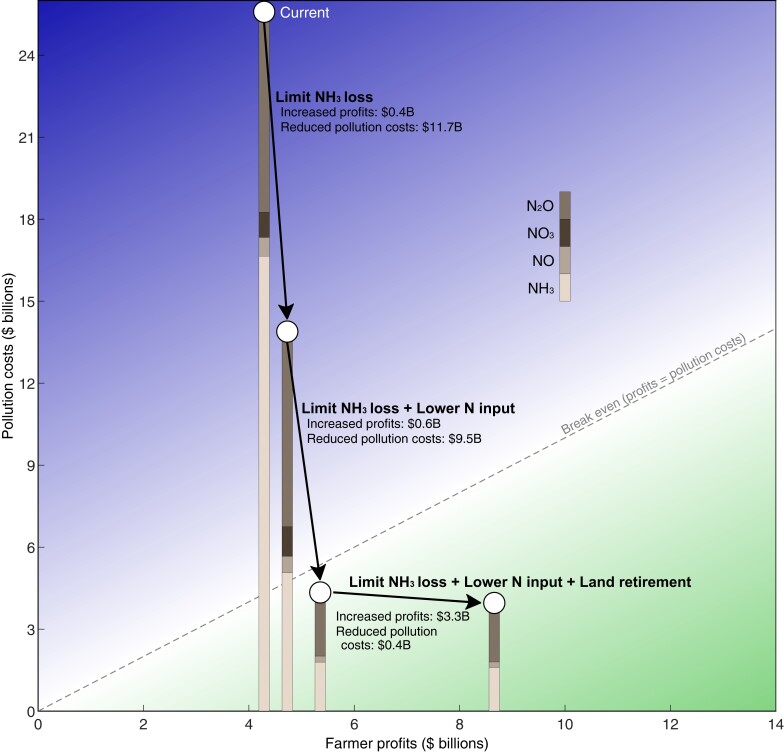
Farmer profits increase and pollution costs decrease across three changes in agricultural practices. Aggregate farmer profits (horizontal axis) and pollution costs (vertical axis) for three changes in agricultural practices. The arrows illustrate the progression of the outcomes from the current scenario to each of the three changes in practices we evaluate. Bars represent pollution costs resulting from each type of emissions. The 45° line shows where profits equal pollution costs with points below and to the right in green showing positive net benefits (profits > pollution costs) and points above and to the left in blue showing negative net benefits (profits < pollution costs).

We find that the overall health and environmental costs of current maize production in the US Midwest exceeds farmer profits by a considerable margin. Annual farmer profits (averaged over 2013–22) from Midwest maize production, which does not include government payments, are $4.3 billion per year (2020$), but health and environmental pollution costs from Midwest maize production are $25.6 billion per year (see Fig. 1 and Table 1). Despite these high pollution costs, Midwestern maize is generally more N efficient with lower pollution costs per unit of maize produced than in other areas in the United States (18).

**Table 1.** Comparing outcomes between current practices and alternatives ($ billions).

	Current	Limit NH_3_ loss	Limit NH_3_ loss + lower N input	Limit NH_3_ loss + lower N input + land retirement
Midwest farmer profits plus government payments	$6.39	$6.83	$7.45	$10.34
* Midwest maize farmer profits* ^a^	*$4.30*	*$4.74*	*$5.37*	*$8.61*
* Government payments received* ^a^	*$2.09*	*$2.09*	*$2.09*	*$2.58* ^b^
Government expenditures	−$2.09	−$2.09	−$2.09	−$2.58^b^
Health and environment costs	−$25.59	−$13.89	−$4.34	−$3.96
				
Subtotal: Midwest farmer profits, government expenditures, and health and environment costs	−$21.28	−$9.15	$1.02	$3.80
				
Change in benefits to consumers and non-Midwest producers of maize from increased maize price	n/a	n/a	−$3.93	−$6.23
* Change in benefits to consumers of Midwest maize* ^a^	*n/a*	*n/a*	−*$3.93*	−*$6.23*
* Change in benefits to consumers of non-Midwest maize* ^a^	*n/a*	*n/a*	−*$18.77*	−*$33.67*
* Change in farmer profits for non-Midwest producers of maize* ^a^	*n/a*	*n/a*	*$18.77*	*$33.67*
				
Total: Farmer profits, government expenditures, health and environment costs, change in consumer benefits	−$21.28	−$9.15	−$2.91	−$2.43
				
Change in social welfare relative to current		$12.14	$18.38	$18.85

^a^The italicized text represents subcategories of the non-italicized text rows above.

^b^This total includes government payments of $1.73 billion to farmers that remain in production, and $0.85 billion in compensation for forgone profits to farmers on retired land.

Of the $25.6 billion in total health and environmental pollution costs, NH_3_ emissions are responsible for 65%. N_2_O emissions, the next largest source of pollution costs, are responsible for 29%. ${\mathrm{NO}}_{3}^{-}$ and NO emissions are responsible for 3.6 and 2.7% of total pollution costs (Fig. 1).

**Fig. 2.**
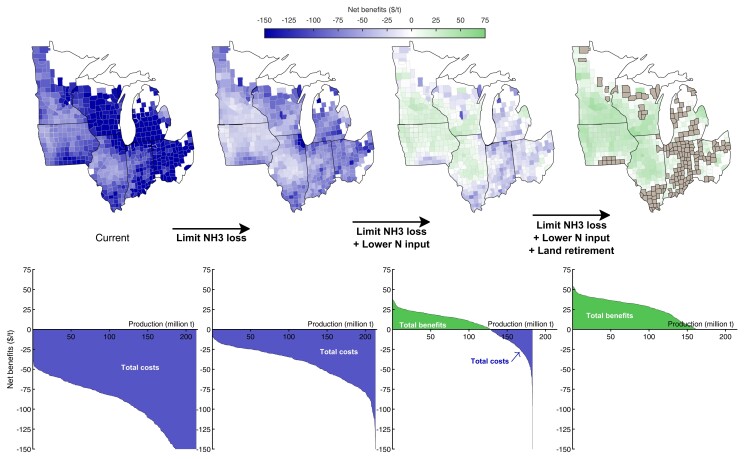
Spatial pattern of improvements in net benefits of maize production. Top: Net benefits (farmer profits minus pollution costs) per t of maize grown in Midwest counties for current practices (left) and three changes in agricultural practices: Limiting NH_3_ loss (center, left); limiting NH_3_ loss plus lowering N input (center, right); and limiting NH_3_ loss plus lowering N input plus land retirement (right). Counties with gray shading (right-most panel) retire land from production. Bottom: Counties ranked from largest to smallest net benefits per t (left to right). The green area above the horizontal line represents the cumulative net benefits of production in counties where profits exceed pollution costs, and purple areas below the horizontal line represent the cumulative net costs in counties where the opposite is true.

Under current practices, pollution costs from maize production exceed the farmer profits in all 593 counties (Fig. 2). Pollution costs by county range from $48 to $688 per metric ton (t) of maize produced. The major determinants of the differences in pollution cost are the rate of ammonia emissions per unit of N fertilizer, and the number of people living downwind exposed to the PM_2.5_ formed from ammonia. Farmer profits, net of government payments, also vary by county, from −$96 to $42 t^−1^ of maize. Profits depend on regional maize yield-response functions (19), which are then adjusted to match the county average yield. Actual farmer profits, which include government payments are higher, but government expenditures must then also be factored into the equation for net benefits (Table 1).

### Limiting NH_3_ loss

N fertilization has different environmental impacts, depending on the N formulation, method of application, and other processes that determine the transport of N into the environment (20). The following intervention shows that the health and environmental impacts of N fertilization can be reduced by changing the application methods and the form of N fertilizer.

One possible intervention is to change the type of N fertilizer to reduce the average rate of NH_3_ losses from N amendments. Under current practices, given the mix of nitrogen fertilizers and quantity of manure applied, we estimate that 8.2% of applied N is lost to the atmosphere as NH_3_, on average. In this intervention, we model a 72% reduction in the estimated NH_3_ loss rate, from 8.2 to 2.3% of N applied. Such a reduction could be achieved in a number of ways. Here, we model the application of anhydrous ammonia in all locations, a commonly used low-NH_3_-loss fertilizer that is injected into the ground (the NH_3_ loss rate we use is an average across three studies (21–23)). In a limited number of areas where anhydrous ammonia may not be effectively applied (24), this low emission rate may also be achieved through a variety of changes to farming practices in addition to switching fertilizer types, including using nitrification inhibitors, incorporating N into the soil after application, and changing fertilizer application timing.

Reducing the NH_3_ emission rate to 2.3% reduces pollution costs by $11.7 billion per year (46%), largely from improved air quality, with the benefits concentrated in downwind dense population centers in the Midwest, and to a lesser extent the Atlantic and New England regions. Limiting NH_3_ losses also leads to slightly lower farmer costs—due here to a lower price of anhydrous ammonia plus the cost of applying fertilizer—however, there may be short-term implementation costs that are not accounted for here. This single change in farming practices greatly improves net benefits across the Midwest. However, pollution costs still exceed farmer profits by $9.1 billion per year (Fig. 1), and all counties still have negative net benefits of producing maize (Fig. 2). N_2_O emissions are largely unchanged with this intervention and represent 51% of the remaining pollution costs (Fig. 1).

### Lowering N input (+ limiting NH_3_ loss)

Lowering the quantity of N fertilizer, in addition to limiting NH_3_ losses, reduces pollution costs further, mainly from fewer emissions of N_2_O. Using estimated maize yield functions, the price of fertilizer, and the pollution costs of adding N fertilizer, we calculate the N application rate that maximizes farmer profit minus health and environmental costs in each county (see Fig. S2). With this calculated rate—which differs from the current situation by raising the cost of N application by including its pollution costs—the quantity of N fertilizer applied per ha across the Midwest is reduced by 63%. A potential mechanism—fraught with political and practical obstacles—for implementing this N application rate is county-specific taxes on fertilizer equal to pollution costs per unit of material. The wider use of best management practices as informed by public extension services may also contribute to this goal.

This intervention reduces pollution costs by $9.5 billion (68%) beyond that achieved by limiting NH_3_ losses alone (Fig. 1). Reducing N application lowers yields by 14.2%. However, it also leads to a 13% increase in farmer profits (increase of $0.6 billion) because buying less fertilizer lowers costs, and the 14.2% decrease in production also leads to a higher price of maize. Using an estimate of the price elasticity of demand (25), we calculate a 12.3% increase in the global price of maize resulting from this reduction in production. The price increase results in a gain to farmer profits but a loss to consumers (Table 1).

Limiting NH_3_ losses and lowering N application rates convert net economic losses for many counties into net gains—the net benefits are positive for 252 of 593 counties—especially in Iowa, Minnesota, central Illinois, and southern Wisconsin (Fig. 2) and profits from maize production exceed pollution costs overall in the Midwest by $1.0 billion per year (Fig. 1). Part of the gains to farmers and from less pollution is offset by buyers of Midwest maize who pay an additional $3.9 billion given the higher price (see Table 1). Overall, social welfare is improved by $18.4 billion compared with current practices, which is the sum of changes in farmer profits, government expenditures, health and environmental costs, and expenditures on maize by consumers (Table 1).

### Land retirement (+ limiting NH_3_ loss + lowering N input)

Retiring land from maize production in areas that are relatively less N efficient and/or that have high pollution costs could further reduce health and environmental costs. In some counties, even with limiting NH_3_ emissions and reducing N application, the environmental costs from maize production still exceed farmer profits. This outcome is more likely in areas that require more N amendment per unit of maize and have higher air pollution costs, often closer to places with greater population density—counties with negative net benefits are, on average, 15% less productive per unit of N input and have 34% higher NH_3_ air pollution costs per unit emission than counties with positive net benefits. Fig. S2 illustrates these factors for a representative county with positive net benefits (left panel) and negative net benefits (right panel). Places with large nearby populations that are exposed to fertilizer-derived air pollution of NH_3_ and NO have high health costs, meaning that retiring land from maize production increases net benefits for society even where maize yields may be high. Halting maize production can be accomplished by fallowing fields or switching to a less N-intensive crop.

Such beneficial land retirements are located largely in Indiana, southern Illinois, Ohio, and Michigan (counties in gray in the top-right panel in Fig. 2). These counties represent 16% of the current land area in the Midwest growing maize. Under current farming practices, these 204 counties produce 14% of maize in the Midwest, generate 19% of pollution costs, and have lower than average profits per ha. Retiring land from maize production in these counties results in a small additional decrease ($0.38 billion) in health and environmental costs in the Midwest (85% lower than under current practices; Fig. 1). To incentivize retirements, we include government compensatory payments of $0.85 billion to farmers for foregone profits. Despite these payments, there are important considerations of inequality and the effect on rural communities from shutting down production. Alternatively, investments through the Environmental Quality Incentives Program could be made to encourage growing of less N-intensive crops with fewer environmental costs.

Overall, retiring land, on top of limiting NH_3_ loss and lowering N input, results in just a small increase in net benefits, $18.9 billion gain versus $18.4 billion gain without land retirement (Table 1). Retiring land increases farmer profits in the Midwest by an additional $3.2 billion (two times higher than under current practices). However, much of the increased profits comes at the expense of consumers who pay a higher price for maize. Consumers of Midwest maize pay an additional $6.2 billion. The increase in farm profits is due to a 22% increase in the global price of maize that occurs from reduced maize production. The price increase boosts farm profits on operating farms far more than the lost profits in counties with land retirements.

## Discussion

Large-scale food production is essential for humanity, but optimal societal outcomes require a balance with environmental quality and a healthy population. At present, the health and environmental costs from maize production in the Midwest far exceed the profit generated. Our analysis of maize production shows that it is possible to achieve dramatic reductions in health and environmental costs while simultaneously increasing farmer profits. These gains are achievable with existing technology and knowledge. While implementing these changes will likely be difficult, our analysis shows that changes in farming practices that use existing technology and known methods, when spread over millions of ha, can yield large gains in environmental quality.

Ammonia emissions from N fertilization, harming human respiratory health, make up the largest share of pollution costs from maize production in the Midwest. Farmers have a variety of options to limit NH_3_ emissions, including switching to low-volatilization N fertilizers, applying urease inhibitors when applying urea, incorporating fertilizer into the soil after application, switching to other crop rotation systems, or changing the timing of fertilizer applications. Here, we modeled the adoption of anhydrous ammonia by all farmers, which on average is less costly than other fertilizers, and is already the most commonly applied synthetic fertilizer in parts of the Midwest. Widespread adoption of anhydrous ammonia could potentially increase water-borne N losses. However, given the large environmental costs of airborne ammonia releases, we estimate that the benefits of reduced air pollution exceed the costs of increased water pollution. Our interventions assume a cessation of manure application on maize fields because of its large ammonia losses, often the result of large quantities of manure application on fields in excess of crop demands for N (26). This would necessitate a major shift in the disposal of manure, or require manure treatment—such as slurry acidification (27)—to make it a low-ammonia-emitting N amendment. We did not include costs associated with alternative treatment or disposal of manure. In Supplemental Information Table S3, we demonstrate that if the quantity of manure were to be held fixed in all interventions, the results and the implications of the model remain largely unchanged.

Our estimates are sensitive to the parameters used in the model. A change in the price of maize greatly influences farmer profits, and there is substantial uncertainty in the value of damages from pollution. Our analysis used well-established, but generally conservative estimates of the pollution-cost coefficients. For human health effects from air pollution, an alternative study (28) estimates twice the rate of mortality from exposure to PM_2.5_ compared with the study we used here (29). For water quality damages from ${\mathrm{NO}}_{3}^{-}$, there is great uncertainty in the valuation of the environmental impacts (30, 31). Our estimates of water quality damages could be many times larger if we included additional categories of impacts (2), potentially leading to recommendations for even larger reductions in N application. We include ${\mathrm{NO}}_{3}^{-}$ damages relating to treatment of groundwater drinking sources (32) and health costs associated with N-contaminated water sources (33), which combined are $0.99 per kg-${\mathrm{NO}}_{3}^{-}$-N released, comprising 3.6% of the total pollution costs—on average, 27% of N applied is lost as ${\mathrm{NO}}_{3}^{-}$-N in the current situation. Including impacts from coastal eutrophication (32), methane-driven climate impacts indirectly arising from eutrophication (34), and hypoxia in the Gulf of Mexico (32) could greatly increase the ${\mathrm{NO}}_{3}^{-}$-related pollution costs. Better characterization of ${\mathrm{NO}}_{3}^{-}$ pollution costs is a crucial area for future work.

The politics of changing agricultural systems often involves difficult tradeoffs and conflicts among groups, some of whom may benefit from the change while others may lose. In our analysis of maize farming however, we found that adopting alternative agricultural practices that improve health and environmental quality can also increase farmer profits. Because farmer's profits increase, we expect that it may be easier politically and economically to adopt alternative production practices that yield health and environmental improvements. States with the largest gains in farmer profits and largest reductions in pollution costs—such as Minnesota, Wisconsin, and Iowa—may be locations to initially target our proposed interventions. Of course, accomplishing any change requires overcoming obstacles and short-term constraints such as contractual obligations, access to inputs, and learning costs. Beyond the actions at the farm level, large-scale changes require engagement by policy makers with other impacted parties—such as fertilizer manufacturers and livestock facilities who purchase maize for feed (35)—and input suppliers, such as large seed companies, who may limit choices available to farmers, leading to suboptimal outcomes for the environment (36, 37).

Our findings do not mean that there are no potential conflicts. In particular, land retirements impose losses on farmers whose land is idled or must switch to other crops, and may have larger implications for rural farming communities. For Midwest maize production, we calculate that the loss in profits to farmers who retire land is $0.85 billion. The Conservation Reserve Program (CRP), an existing program with annual outlays of ∼$3.5 billion (38) that pays farmers to retire land, could be used to fund compensation to farmers for strategic land retirements.

Decreased production, which results in increased prices to consumers—both in the maize market and animal protein markets that rely heavily on maize—is another potential source of conflict. The maize price rise increases farmer profits and is more reflective of the full costs of crop production by including the costs of pollution; however, agricultural price increases have a detrimental impact on consumers, especially those in low-income countries. When applying large-scale changes to agricultural systems, including agricultural commodities on which the poorest populations globally depend, the distributional impacts of food price increases (or price variability) are especially important to consider. The recent increase in the price of agricultural commodities and potential impacts on global food shortages caused by the conflict in Ukraine amply illustrates the negative consequences of a reduction in supply of key agricultural commodities (39). Because most US maize production is used as animal feed, the price increases could result in higher prices in animal protein markets. In response to a price increase induced by reduction in maize production in the Midwest, farmers elsewhere may increase maize production. This supply response could dampen the price increase, limiting the negative impact on consumers, but would increase health and environmental costs elsewhere, including the potential to contribute to large releases of stored carbon if additional land is brought into production (40). If instead, farmers switch other crops to maize then there may be price increases for other crops.

Due to data limitations, our work was based on county averages. Consideration of variation within a county could allow for greater improvements in net benefits—for example, by considering retirements only on the least-productive land within a county, opposed to an all-or-nothing decision for the whole county.

Our modeling approach, seeking alternative production practices that reduce health and environmental impacts while maintaining profits for farmers, could be applied to other regions and crops. N is the major limiting nutrient for growth of many important crops (41) and N fertilizer use worldwide may be 70–100% greater in 2050 than in 2000 (42), so the management and environmental effects of N discussed here are likely to be of increasing global importance. Obtaining the necessary data is the main obstacle in applying our methods elsewhere, especially as a global version of the air-quality model used here has recently been published (43). Location-specific estimates of crop yield curves and the effect of nitrogen losses were available for our current application, but may not be widely obtainable. The data requirements to model changes in environmental impacts and profits from changes in practices are substantially greater than estimating baseline impacts and profits.

There are additional changes in production practices that could be analyzed, such as inclusion of buffer strips, tiling practices, cover crops, other field amendments, or changes in residue management. We focused on N management based on a previous analysis that indicated that nitrogen-related health and environmental costs are the largest share of pollution costs in the Midwest (18). In addition, we considered only changes within the current dominant maize production system—and assumed the continued reliance on a two-year maize-soybean rotation—but it is also possible to expand the analysis to consider whether more fundamental shifts in the agricultural system would be more beneficial.

Our analysis highlights that it is possible to identify changes in practices that lead to net gains for both farmers and those impacted by agricultural pollution. Agriculture is a significant contributor to pollution in many countries, but it has proven difficult to address both because the source of pollution is spread among many farmers rather than concentrated at a small number of point sources, and policies that do not reward farmers economically are politically unpopular. It becomes far easier to reduce pollution when there are easy-to-adopt alternatives that are profitable for farmers. In our application, this mutual gain was possible because the proposed changes in some cases reduced production costs or resulted in an increase in the commodity price.

## Materials and methods

We created a model to examine maize production and the effects of changes in N use on yields and pollution. Our analysis simulates a single year of maize production, representing averages across 2013–22. The unit of analysis is at the county level, across seven states in the US Midwest Corn Belt (Illinois, Indiana, Iowa, Michigan, Minnesota, Ohio, and Wisconsin). The model combines several components as follows: (i) sub-state maize yield-response functions (to simulate maize production based on N amendments), (ii) N mass-balance equations to account for nitrogen additions and subtractions on farmland, and (iii) estimates of pollution costs from different forms of N releases into the environment, including location-specific estimates of air pollution impacts. In the N mass-balance equations, additions make N available for the maize plant, affecting yields and farmer profits, and subtractions are from N uptake by the maize plant and from N losses to pollution leading to health and environmental costs. The model compares farmer profits with the health and environmental costs of N pollution to characterize the current situation, and three proposed interventions in N management.

The model assumes an annual N balance such that the amount of N added to the field or soil is subtracted in some form. While we only model the outcome of a single maize production year, we assume that the land is used in a two-year maize-soybean rotation. The following sections lay out the parameters and the relationships between variables used in the model.

### N additions

Additions of N include amendments in the forms of synthetic N fertilizer ($N^{\mathrm{fert}}$) and manure ($N^{\mathrm{manure}}$), and N in other forms ($N^{\mathrm{other}}$)—these other forms include atmospheric deposition of N, nonsymbiotic fixation of N, and N in the maize seed (44). $N^{\mathrm{fert}}$ and $N^{\mathrm{manure}}$ are decision variables in the model, representing the amount of N applied to the field ($N^{\mathrm{applied}}=N^{\mathrm{fert}}+N^{\mathrm{manure}}$), while other forms of N are treated as a fixed constant, $N^{\mathrm{other}}$ = 16.4 kg N/ha (44).

Under current practices, data on the rate of $N^{\mathrm{fert}}$ per ha is at the state level (45) applied uniformly to each county in the state. $N^{\mathrm{manure}}$ is also based on a state-level data, calculated based on the share of maize ha that apply manure, and the rate per manured ha (see Table S1) (46). The N concentration in manure, as applied, is based on the share of manure by animal type (beef, dairy, pig, and poultry) and system of management (liquid and dry) at the state level, and the percentage of N in each system/type (47). The share of N applied to maize from manure varies substantially across the states we examined—as little as 4% of N applied is from manure in Illinois and Ohio, and as much as 36% of N applied is from manure in Wisconsin.

To characterize the current practices scenario, we simulate separately those farms that apply only synthetic N fertilizer and those that use both synthetic fertilizer and manure. In both cases, we assume that the total quantity of N/ha applied within the state is the same—except for in Iowa in which the quantity of N from manure/ha is greater than the state average N/ha. Table S2 shows the share of maize ha that apply only synthetic fertilizer (and the rate of N application), and the share of maize ha that apply both synthetic and manure N (and the rate of application for each) for the current scenario by state. Across our study area under current practices, we estimate that 15% of N applied is from manure. We assume that synthetic N fertilizer is applied in three possible forms: anhydrous ammonia (AA), urea (U), and urea-ammonium nitrate solution (UAN). To characterize current practices, we use an estimate of the share of N applied by each of the three types at the county level (for all crops) (48).

### N subtractions

Nitrogen subtractions include uptake by the maize plant in the grain and emissions into the environment. N uptake is separated into two parts as follows: N in the grain which is removed from the field, and N in the other parts of the plant which remain in the field. We define N in the grain as $N^{\mathrm{up}}=\beta^{\mathrm{up}}Y$, which is proportional to the estimated yield (*Y*), where the proportion of N in the maize grain is $\beta^{\mathrm{up}}$ = 0.0115 (49). N in the rest of the plant is not explicitly modeled because we assume that the maize plant litter is left in the field and eventually incorporated back into the soil.

#### Yield

Maize yield-response curves, as a function of N applied, are adopted from an Iowa State N management calculator (19), based on site estimates across several states. Separate yield-response curves are available at various spatial resolutions in each state (e.g. five regions for Indiana, two regions for Iowa, three regions for Illinois, three regions for Wisconsin, and the entire state for Minnesota, Michigan, and Ohio). For each county, *i*, we use the following functional form to approximate the relationship in the yield calculator:


$$Y_{i}=\frac{Y_{i}^{\max}}{100}\left[\mathrm{bas}e_{i}+\gamma_{i}\mathrm{atan}\left(\frac{N_{i}^{\mathrm{applied}}}{\delta_{i}}\right)-\phi_{i}N_{i}^{\mathrm{applied}}\right],$$

where $Y_{i}$ is yield in kg/ha, and $Y_{i}^{\max}$ is the maximum yield for each county given a high input of N. We estimate this value for each county by solving the above equation for $Y_{i}^{\max}$, and using county data on yields (the 10-year average for 2013–22) (50) and the current practices quantity of $N^{\mathrm{applied}}$. The parameter $\mathrm{bas}e_{i}$ is the estimated yield with no N applied, and $\gamma_{i}$, $\delta_{i}$ and $\phi_{i}$ are estimated parameters.

#### N emissions

The remaining N losses are as emissions which are used to estimate pollution costs. Pollution costs are estimated by applying N-loss coefficients to calculate emissions, and marginal pollution-cost coefficients to translate emissions into monetary costs. We model the emission of N in five forms as follows: N_2_, N_2_O, NO, NH_3_, and ${\mathrm{NO}}_{3}^{-}$. We assume that N_2_, which is not harmful, is emitted as a fixed constant, $N^{N2}$ = 2.61 kg N/ha (44).

Next, N_2_O, NO, and NH_3_ are emissions as a function of the quantity of $N^{\mathrm{applied}}$. For N_2_O and NO emissions, we follow the IPCC framework (51). Emissions of NO are a fixed proportion of $N^{\mathrm{fert}}$ and $N^{\mathrm{manure}}$, with emission rates of $\beta^{\mathrm{NO}}$ = 0.005 (52) and $\beta^{\mathrm{NO}\text{-}m}$ = 0.01 (51), respectively ($N_{i}^{NO}=\beta^{\mathrm{NO}}N_{i}^{\mathrm{fert}}+\beta^{\mathrm{NO}\text{-}m}N_{i}^{\mathrm{manure}}$). In the IPCC framework, N_2_O emissions are 1% of total available N, a quantity that includes N amendments and N present in crop biomass. We use an estimate from Decock (53) in which N_2_O emissions are a fixed proportion of $N^{\mathrm{applied}}$ ($\beta^{N2O}=0.017$ and $N_{i}^{N2O}=\beta^{N2O}N_{i}^{\mathrm{applied}}$).

Emissions rates of NH_3_ are modeled as a fixed proportion of $N^{\mathrm{applied}}$, with different rates by fertilizer type. The NH_3_ volatilization rates are the average of estimates from three sources (21–23). The rate of N loss as NH_3_ for each type is: $\beta^{\mathrm{NH}3\text{-}\mathrm{AA}}$ = 0.023 for anhydrous ammonia, $\beta^{\mathrm{NH}3\text{-}U}$ = 0.17 for urea, and $\beta^{\mathrm{NH}3\text{-}\mathrm{UAN}}$ = 0.07 for urea-ammonium nitrate solution. Emissions of NH_3_ from manure are estimated as a fixed proportion, 35% (54), of total ammoniacal N (TAN: N in the forms of ammonium and ammonia) in the manure. We estimate TAN at the state level based on the manure by animal type and management system (47), leading to state-specific emission rates, $\beta^{\mathrm{NH}3\text{-}m}$. N lost as NH_3_ is $N_{i}^{\mathrm{NH}3}=N_{i}^{\mathrm{fert}}(s_{i}^{\mathrm{AA}}\beta^{\mathrm{NH}3\text{-}\mathrm{AA}}+s_{i}^{U}\beta^{\mathrm{NH}3\text{-}U}+s_{i}^{\mathrm{UAN}}\beta^{\mathrm{NH}3\text{-}\mathrm{UAN}})+N_{i}^{\mathrm{manure}}\beta^{\mathrm{NH}3\text{-}m}$, where the $s_{i}$ are the shares of each fertilizer type by county.

Leaching of ${\mathrm{NO}}_{3}^{-}$ is assumed to occur after N uptake and other emissions, and serves to balance the N equation. This is a simplification of nitrate leaching and likely overestimates the annual nitrate losses as N can accumulate on cropland and leach over many years (55). We define $N^{\mathrm{NO}3}$ as the difference between N additions and subtractions:


$$N^{\mathrm{NO}3}=(N^{\mathrm{applied}}+N^{\mathrm{other}})-(N^{\mathrm{up}}+N^{N2}+N^{\mathrm{NO}}+N^{N2O}+N^{\mathrm{NH}3}).$$

Following the IPCC framework (51), we include indirect emissions of N_2_O resulting from emissions of NH_3_, NO, and ${\mathrm{NO}}_{3}^{-}$. These emissions do not alter the N-balance equation above as all calculations are made after determining the emissions of each pollutant. Using IPCC coefficients, we apply 1% indirect emissions from NH_3_ and NO, defined as $\beta_{1}^{\mathrm{ind}}$, and 0.75% indirect emissions from ${\mathrm{NO}}_{3}^{-}$, defined as $\beta_{2}^{\mathrm{ind}}$. Indirect N_2_O emissions are


$$N^{N2O\text{-}\mathrm{ind}}=\beta_{1}^{\mathrm{ind}}(N^{\mathrm{NH}3}+N^{\mathrm{NO}})+\beta_{2}^{\mathrm{ind}}N^{\mathrm{NO}3}.$$

Total emissions for each county are calculated by converting the N emissions (net of any indirect loss or gain) of each pollutant from units of N to units of each compound. These emissions are defined as $E_{i}^{N2O}$, $E_{i}^{\mathrm{NO}}$, $E_{i}^{\mathrm{NH}3}$, and $E_{i}^{\mathrm{NO}3}$.

### Pollution costs

Emissions are translated into monetary pollution costs by applying available pollution-cost coefficients (see summary of per unit pollution costs in Table 2). N_2_O emissions contribute to global climate change and pollution costs are calculated according to an N_2_O adjustment (56) to an estimate of the social cost of carbon (SCC) from Rennert et al. (57). We use a $185 SCC for CO_2_ with the 2% discount rate for 2020 emissions. After adjusting for inflation to 2020$ and for N_2_O emissions, we get a pollution-cost coefficient of $\lambda^{N2O}$ = $66.81/kg N_2_O.

**Table 2.** Per unit pollution costs (per unit of each compound, and per unit of N).

	Per unit pollution cost	
Pollutant	$/kg	$/kg N
N_2_O	66.8	105.0
${\mathrm{NO}}_{3}^{-}$	0.22	0.99
NH_3_	[31.0, 70.0]	[37.7, 85.1]
NO	[13.8, 18.3]	[29.6, 39.2]

A single value was applied to emissions from any county for N_2_O and ${\mathrm{NO}}_{3}^{-}$. A county-specific value was applied to emissions of NH_3_ and NO—the values shown in the table are the interquartile range across the counties.

For leaching of ${\mathrm{NO}}_{3}^{-}$, we use estimates of the cost of treating drinking-water sources (32) and the health costs associated with cancer risk from drinking N-contaminated water (33). The pollution-cost coefficients are converted into 2020$ and are equal to $0.19/kg ${\mathrm{NO}}_{3}^{-}$-N for treatment and $0.80/kg ${\mathrm{NO}}_{3}^{-}$-N for health costs ($\lambda^{\mathrm{NO}3}$ = $0.22/kg ${\mathrm{NO}}_{3}^{-}$ after converting to units of ${\mathrm{NO}}_{3}^{-}$). The spatial impacts of leaching of ${\mathrm{NO}}_{3}^{-}$ vary by location, but this variability is complex and uncertain (12), and are therefore not included in this analysis.

Emissions of NH_3_ and NO can chemically react in the atmosphere to form particulate ammonium and particulate nitrate (58), which are components of fine particulate matter (PM_2.5_). PM_2.5_ concentrations, when inhaled, increase the risk of adult premature mortality (59, 60). Agricultural practices are by far the largest source of NH_3_ emissions, both globally (61) and in the United States (7). NH_3_ is the most abundant base in the atmosphere, and it rapidly neutralizes ambient acids to form particulate ammonium (58), which can travel far in the atmosphere and affect human health. Factors other than emission sources (e.g. proximity to pollution centers, meteorology) further affect the extent to which NH_3_ affects human health (18). The mortality effect of emissions varies greatly depending on the location of emission (62). We use air pollution coefficient estimates from InMAP, an air-quality model that isolates the effect of emissions at each location (63, 64) (see Fig. S1 for county-specific coefficients for NH_3_ emissions). The evaluation of our modeling approach, including the air-quality model InMAP (that drives many of our conclusions) is documented in several publications, including against observed pollutant concentrations (63, 65) and against other models (63, 66). Against measurements, InMAP underpredicts total PM_2.5_ concentrations (mean fractional bias [MFB]: −38%; mean fractional error [MFE]: 47%) and particulate ammonium (MFB: −50%; MFE: 53%). Furthermore, InMAP reproduces WRF-Chem-derived particulate ammonium concentrations fairly well for a range of policy scenarios (population-weighted MFB: −47%; MFE: 93%).

To calculate pollution costs of air quality changes, we convert changes in PM_2.5_ concentrations to premature mortality (29), and apply the US EPA's value of a statistical life figure of $9.4 million to convert premature mortality into monetary pollution costs (67). We only include premature mortality impacts from air pollution, excluding other potential impacts such as morbidity and lost work days. The pollution-cost coefficients $\lambda_{i}^{\mathrm{NO}}$ and $\lambda_{i}^{\mathrm{NH}3}$ vary by county based on the impact of emissions on downwind populations, according to the InMAP model.

### Farm profits

Profits per ha to the farmer are equal to the revenue from crop sales, minus the costs of inputs


$$\pi_{i}=pY_{i}-wN_{i}^{\mathrm{fert}}-C_{i}^{\mathrm{fert}\;\mathrm{app}}-C_{i}^{\mathrm{non}\text{-}\mathrm{fert}}.$$

Revenue is yield times the maize price (*p*, the 10-year inflation adjusted average price from 2013–22) (50). Costs are separated into costs of buying and applying fertilizer, and non-fertilizer costs. The cost of fertilizer is subtracted off the regional total maize production costs per ha (68) to get all non-fertilizer costs. The cost of buying fertilizer is based on the unit price of fertilizer, *w* (the average of the bi-weekly price from 2013–22 (69)), and the quantity purchased. The cost to apply fertilizer, $C^{\mathrm{fert}\;\mathrm{app}}$, is specific to each type of fertilizer (70)—$32.37 per ha cost of injection for anhydrous ammonia, $13.22 per ha cost of spreading urea, and $16.19 per ha cost of spraying liquid UAN. Farmer profits refer to the net returns from these direct revenues and costs described above, and do not include government subsidies. Government payments, which are the state average payments per ha over our study period, are also included in the model as an additional source of revenue for farmers and as an expenditure for the government (see Table 1).

### Scenarios and interventions

In each scenario, we calculate, for every county, the farmer profits, pollution costs, government payments and expenditures, and net social welfare (see Table 1). These are calculated in total, per ha, and per t of maize (as displayed in Fig. 2).

#### Current practices

For every county, the model is run for each fertilizer type and with and without manure, using the current practice quantity of $N^{\mathrm{applied}}$. For each county, we calculate a weighted average of each model run, where the weights are the share of each fertilizer type and manure used.

#### Limit NH_3_ loss

For the limit NH_3_ loss intervention, the current practice quantity of $N^{\mathrm{applied}}$ is used, but instead of the current practice mix of fertilizer types and manure, all farms use anhydrous ammonia. The price differences between the fertilizers are relatively small, and other factors—including regional price differences, application costs, fertilizer timing flexibility, equipment requirements and safety, and soil toxicity and pH—likely explain the current mix of fertilizer types used.

#### Limit NH_3_ loss plus lower N fertilizer inputs

In addition to switching to anhydrous ammonia, this intervention uses the rate of synthetic N fertilizer per ha that maximizes social net benefits. This fertilizer rate, $N_{i}^{⋆}$, solves:


$$\underset{N_{i}}{\max}\;\{SW_{i}=\pi_{i}-\lambda^{N2O}E_{i}^{N2O}-\lambda_{i}^{\mathrm{NO}}E_{i}^{\mathrm{NO}}-\lambda_{i}^{\mathrm{NH}3}E_{i}^{\mathrm{NH}3}-\lambda^{\mathrm{NO}3}E_{i}^{\mathrm{NO}3}\}.$$

The quantity $N_{i}^{⋆}$ that maximizes social welfare ($SW_{i}$) equates the marginal revenue of additional fertilizer with the entire social cost of fertilizer, which includes the price of fertilizer, *w*, and the marginal pollution costs of each additional unit of fertilizer (see Fig. S2 for an illustration with two counties).

#### Limit NH_3_ loss plus lower N fertilizer inputs plus land retirement

There are counties in our model such that the pollution costs exceed farmer profits even with limited NH_3_ losses and at the fertilizer rate $N_{i}^{⋆}$. In these counties, social welfare is improved by retiring these counties from maize production—and the resulting social welfare in these counties is zero.

### Increase in maize price

In the last two interventions, with lower N fertilizer use and land retirements, maize production in the Midwest is reduced. In the model, we simulate that this maize reduction leads to an increase in the global price of maize. An increase in the maize price increases farm profits, and makes the social welfare in some counties that would be negative given the initial price, positive with the higher price. To determine the ultimate increase in the price of maize, we use an estimate of the global elasticity of demand for maize (25), and use an iterative process to find the solution—running the model several times, calculating the price and social welfare of every county after each run, and continuing this process until the price does not change between runs. We assume a constant-price elasticity of demand functional form:


$$p(Q)={\left(\frac{Q}{k}\right)}^{-\frac{1}{\varepsilon }}$$

where *Q* is the global quantity of maize (in kg), *k* is a constant, and ε = 0.244 is the price elasticity of demand. We calculate *k* = 6.16 × 10^10^, using the initial price and the global quantity of maize (average between 2013 and 2022) (71). We also calculate the lost consumer surplus for buyers of maize from the change in production (i.e. the welfare lost by buyers of the maize that is no longer produced and the higher price):


$$\Delta CS=\int_{Q^{0}}^{Q^{⋆}}\;(p(Q)-p^{0})dQ.$$

## Supplementary Material

pgad319_Supplementary_Data

## Data Availability

The datasets generated for the current study are available as Supplementary Files.
