# Supplementary material for: Managing nitrogen in maize production for societal gain
Source: PNAS Nexus. 2023 Oct 24;2(10):pgad319. doi: 10.1093/pnasnexus/pgad319 (PMC10597588; doi:10.1093/pnasnexus/pgad319)
Supplement: pgad319_Supplementary_Data [file pgad319_supplementary_data.zip › PNASNEXUS-PNASNEXUS-2023-00310R-s01.docx]

**
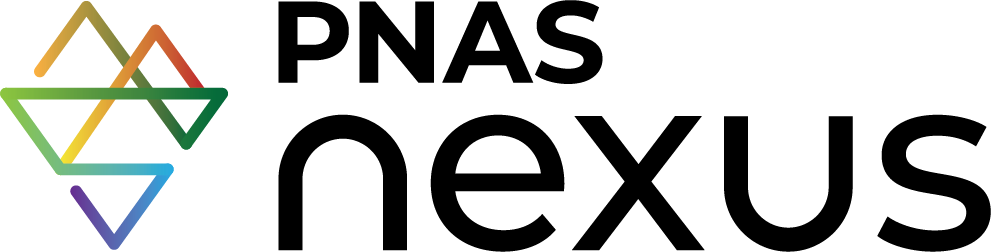
**

**Supplementary Information for**

Managing nitrogen in maize production for societal gain

Andrew L. Goodkind^1,*^, Sumil K. Thakrar^2,3^, Stephen Polasky^3^, Jason D. Hill^2^, David Tilman^4^

^1^ Department of Economics, University of New Mexico, Albuquerque, NM 87131, USA.

^2^ Department of Bioproducts and Biosystems Engineering, University of Minnesota, St. Paul, MN 55108, USA.

^3^ Department of Applied Economics, University of Minnesota, St. Paul, MN 55108, USA.

^4^ Department of Ecology, Evolution, and Behavior, University of Minnesota, St. Paul, MN 55108, USA.

* Correspondence to: agoodkind@unm.edu

**This PDF file includes:**

Supporting text

Figures S1 to S2

Tables S1 to S3

**Supplemental Information**

**Fixed manure scenario**

We evaluate an alternative scenario in which the quantity of manure applied to maize fields remains fixed across all possible interventions. The justification for such a scenario is that the animal operations that produce manure largely requires the use of crop fields to dispose of the manure. This alternative scenario limits the decision makers to only choosing the quantity and type of synthetic N fertilizer, while maintaining the current practices quantity of manure on the fraction of fields that apply manure (see Table S1). This scenario provides one extreme, in which there is no reduction in manure, while the results presented in the main manuscript provide the other extreme, in which no manure is applied for any of the interventions due to the vary large quantity of ammonia emissions.

For this fixed-manure scenario, the costs of acquiring and applying the manure and the health and environmental costs resulting from the application of the manure are not included in the decision-maker’s problem, and are excluded from the results in Table S3. These are fixed costs that are outside of the decision-maker’s control, and most importantly impacts the land retirement decision by only counting the benefits and costs of the decisions within the control of the farmer. The N from the manure applied is included in the maize yield function and does impact the decision regarding the quantity of synthetic N to apply.

The results from the fixed-manure scenario are shows in Table S3 which can be compared against Table 1. The results are broadly similar to the results in Table 1. The largest difference is that the health and environmental costs under current practices are $11.29 billion more than in the fixed-manure scenario. This is due entirely to the exclusion of the health and environmental costs from the fixed-manure calculation. In the three intervention scenarios, the health and environmental costs are slightly less with manure fixed than in the main-manuscript results. Farmer profits are higher in the fixed-manure scenario, and this is due to the need to replace effectively free manure with synthetic N in the main-manuscript results. In both of the last two interventions (*Limit NH_3_ loss plus lower N fertilizer inputs* and *Limit NH_3_ loss plus lower N fertilizer inputs plus land retirement*) the total quantity of N applied is greater (by 12–13%) in the fixed-manure scenario compared with the main results. In the final intervention including land retirements, one fewer county shuts down production (203 versus 204) and the maize price increase is slightly less (20% versus 22%) in the fixed-manure scenario compared with the main results.

**Supplemental figures and tables**


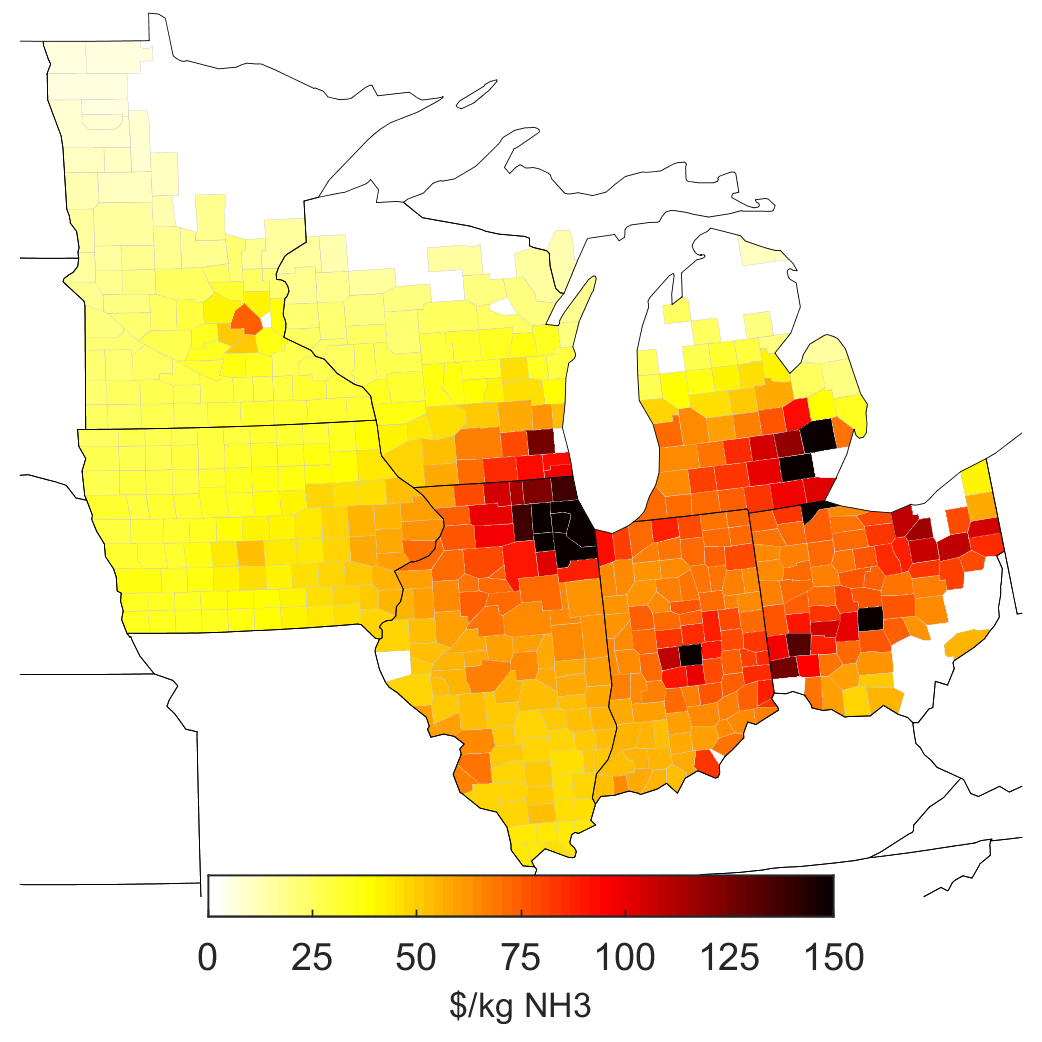


**Fig. S1.** NH_3_ pollution cost coefficient (or marginal pollution cost) varies substantially by emission location. The figure represents the monetary pollution costs from premature mortality per kg of NH_3_ emitted in any location. This does not show the location of where costs occur, rather the magnitude of the cost attributable to the source of emissions.


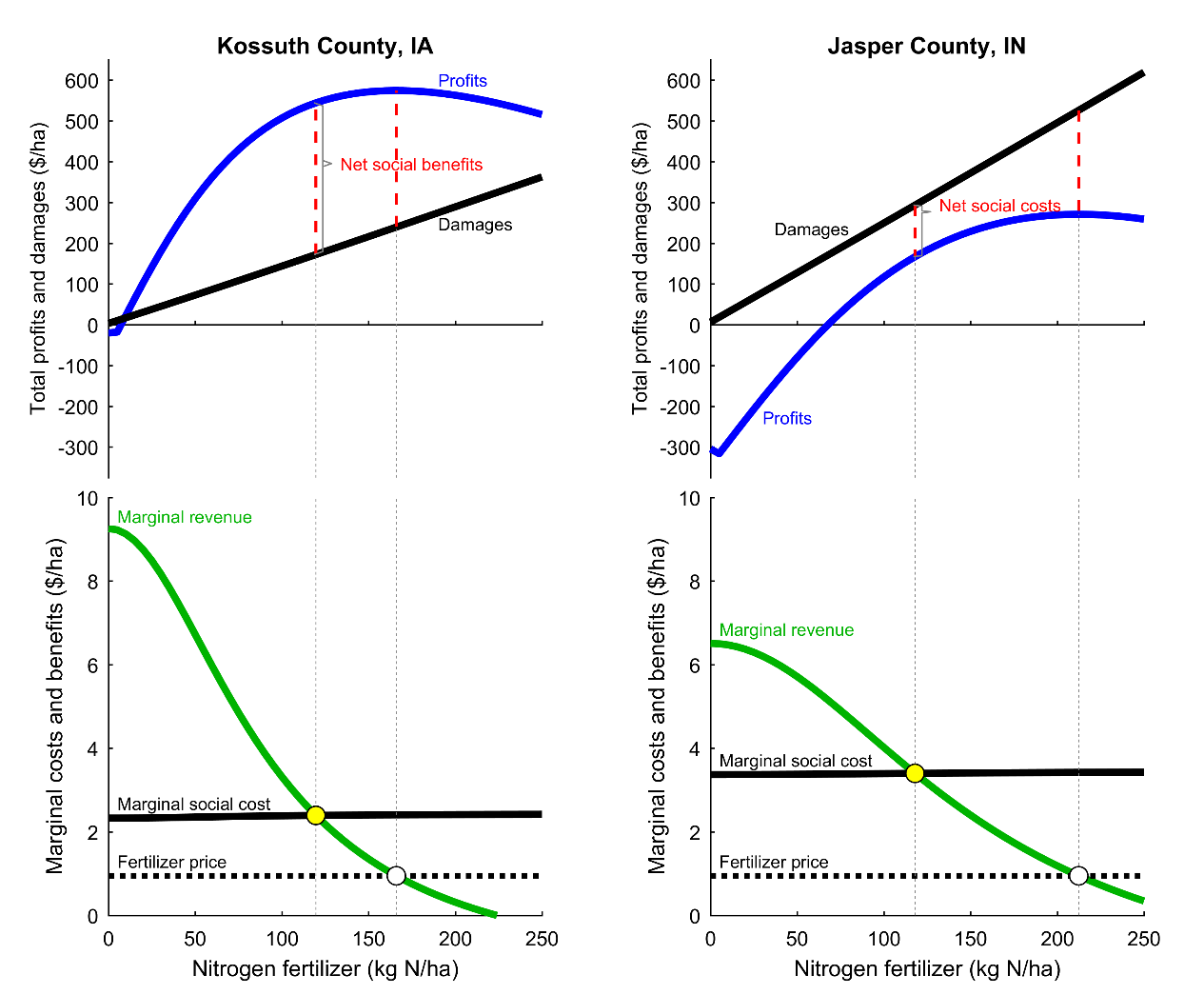


Fig. S2. The top panels show farmer’s profits (blue line) and pollution costs (black line) with different quantities of N fertilizer application per hectare. The bottom panels show the incremental benefits to the farmer (green line) and incremental costs of using an additional kg of fertilizer (black lines) which are the determining factors in the decision on how much to apply. Farmer profits are maximized when the additional revenue from higher yields of applying more fertilizer just equals the price of fertilizer, represented by the white dot. When we account for the environmental damages of applying additional fertilizer, the socially preferred rate is found where the additional revenue to the farmer just equals the combined marginal social costs (fertilizer cost plus pollution cost), represented by the yellow dot.

Table S1. State manure application rates.

| **State** | **% of maize ha applying manure** | **Rate of manure (t/ha)** | **N% of manure** | **kg N per manured ha** |
| --- | --- | --- | --- | --- |
| IL | 6.6% | 17.4 | 0.66% | 115 |
| IN | 8.7% | 19.2 | 0.54% | 104 |
| IA | 16.5% | 31.3 | 0.77% | 242 |
| MI | 18.6% | 28.5 | 0.51% | 144 |
| MN | 27.6% | 36.6 | 0.35% | 128 |
| OH | 10.1% | 14.1 | 0.55% | 78 |
| WI | 50.0% | 44.6 | 0.32% | 141 |

**Table S2.** **N from synthetic fertilizer and manure (kg/ha).**

|  | **Synthetic fertilizer only** | | **Both synthetic and manure fertilizer** | | | |
| --- | --- | --- | --- | --- | --- | --- |
| **State** | **Share of maize ha** | **Synthetic**  **kg N/ha** | **Share of maize ha** | **Manure**  **kg N/ha** | **Synthetic**  **kg N/ha** | **Total**  **kg N/ha** |
| IL | 93.4% | 197 | 6.6% | 115 | 82 | 197 |
| IN | 91.3% | 185 | 8.7% | 104 | 81 | 185 |
| IA | 83.6% | 181 | 16.5% | 242 | 0 | 242 |
| MI | 81.4% | 183 | 18.6% | 144 | 39 | 183 |
| MN | 72.4% | 183 | 27.6% | 128 | 55 | 183 |
| OH | 89.9% | 202 | 10.1% | 78 | 124 | 202 |
| WI | 50.0% | 193 | 50.0% | 141 | 52 | 193 |

**Table S3. Current practices and alternatives ($ billions) with the quantity of manure fixed.**

|  | Current | Limit NH_3_ loss | Limit NH_3_ loss + Lower N input | Limit NH_3_ loss + lower N input + land retirement |
| --- | --- | --- | --- | --- |
| Midwest farmer profits plus government payments | $4.41 | $4.71 | $6.57 | $9.08 |
| *Midwest maize farmer profits*^b^ | *$2.32* | *$2.62* | *$4.48* | *$7.38* |
| *Government payments received*^b^ | *$2.09* | *$2.09* | *$2.09* | *$1.70^a^* |
| Government expenditures | −$2.09 | −$2.09 | −$2.09 | −$2.58^a^ |
| Health and Environment costs ^c^ | −$14.30 | −$7.54 | −$3.79 | −$2.59 |
| Subtotal: Midwest farmer profits, government expenditures, and health and environment costs | −$11.98 | −$4.92 | $0.69 | $3.96 |
| Change in benefits to consumers and non-Midwest producers of maize from increased maize price | n/a | n/a | −$3.79 | −$5.85 |
| *Change in benefits to consumers of Midwest maize*^b^ | *n/a* | *n/a* | −*$3.79* | −*$5.85* |
| *Change in benefits to consumers of non-Midwest maize*^b^ | *n/a* | *n/a* | −*$17.95* | −*$30.19* |
| *Change in farmer profits for non-Midwest producers of maize*^b^ | *n/a* | *n/a* | *$17.95* | *$30.19* |
| Total: Farmer profits, government expenditures, health and environment costs, change in consumer benefits | −$11.98 | −$4.92 | −$3.10 | −$1.89 |
| Change in social welfare relative to current |  | $7.06 | $8.89 | $10.09 |
| ^a^ This total includes government payments of $1.70 billion to farmers that remain in production, and $0.84 billion in compensation for forgone profits to farmers on retired land.  ^b^ The italicized text represents subcategories of the non-italicized text rows above.  ^c^ The health and environmental costs of manure application are excluded from the results in the table. | | | | |
